# Supplementary material for: Multi-metric evaluation and parametric optimization of stochastic gradient boosting machines for genomic prediction and selection in wheat (Triticum aestivum) breeding
Source: G3 (Bethesda). 2026 May 23;16(7):jkag127. doi: 10.1093/g3journal/jkag127 (PMC13334169; doi:10.1093/g3journal/jkag127)

Split-mean benchmark comparison by trait and performance metric

Points are split means; black diamonds show mean +/- 1 SE across matched splits

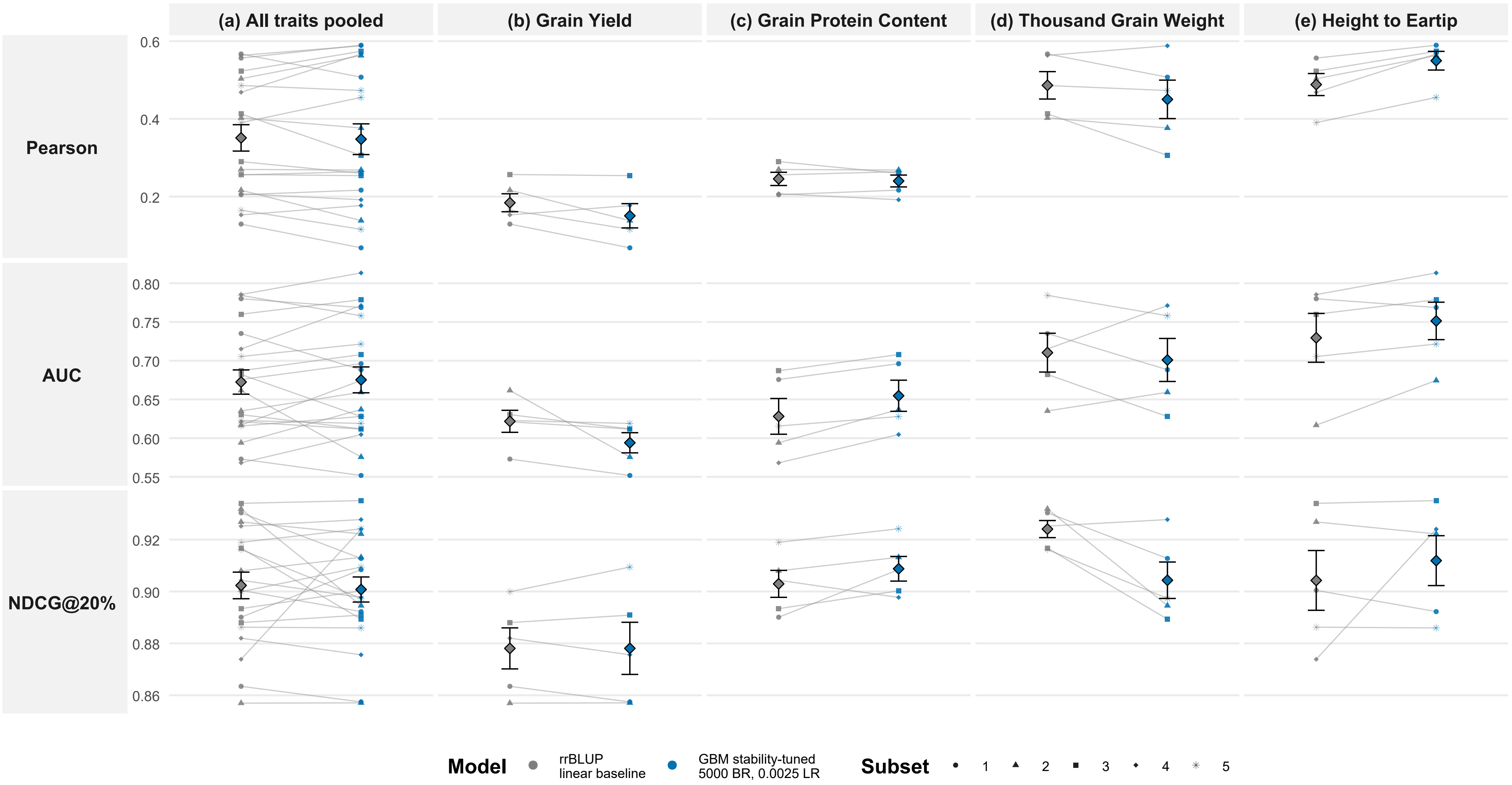

Supplement: jkag127_Supplementary_Data [file jkag127_supplementary_data.zip › Fig._S6_G3-2026-406682.pdf]
